# Garbage in, toxic data out: a proposal for ethical artificial intelligence sustainability impact statements

**DOI:** 10.1007/s43681-022-00221-0

**Published:** 2022-10-20

**Authors:** Ronny Bogani, Andreas Theodorou, Luca Arnaboldi, Robert H. Wortham

**Affiliations:** 1 Department of Law, University of Edinburgh, Edinburgh, UK; 2 Department of Computer Science, Umeå University, Umeå, Sweden; 3 School of Informatics, University of Edinburgh, Edinburgh, UK; 4 Department of Electrical Engineering, University of Bath, Bath, UK

**Keywords:** AI, Ethics, Sustainability, Data lifecycle, Impact statements

## Abstract

Data and autonomous systems are taking over our lives, from healthcare to smart homes very few aspects of our day to day are not permeated by them. The technological advances enabled by these technologies are limitless. However, with advantages so too come challenges. As these technologies encompass more and more aspects of our lives, we are forgetting the ethical, legal, safety and moral concerns that arise as an outcome of integrating our lives with technology. In this work, we study the lifecycle of artificial intelligence from data gathering to deployment, providing a structured analytical assessment of the potential ethical, safety and legal concerns. The paper then presents the foundations for the first ethical artificial intelligence sustainability statement to guide future development of AI in a safe and sustainable manner.

## Introduction

Aiming to address the increasing demand for data presented by artificial intelligence (AI), this paper proposes adopting an ethical artificial intelligence sustainability impact statement (EAISIS) through the various stages of the artificial intelligence development cycle (AIDC). The following analysis treats data as a limited resource requiring sustainable exploitation and management. Following a brief discussion about sustainability and AI, the different stages of the AIDC and accompanying factors for an ethical impact evaluation are addressed through a set of proposed questions. These inquiries are intended to identify, disclose, and address the data’s toxicity level, or potential for ethical harm. The proposed approach to data as a public good both benefits the digital environment and conserves natural resource demand in the real world, i.e. In real life (IRL). Data are currently a commodity, commanding prices higher than oil within a booming data brokerage industry [1]. As with other commodities, derivative markets will form with the price for data lowering proportionally to its level of impurity, or debasement—for data, we can associate this ‘debasement‘ with increased risk of ethical harm. Through using an EAISIS approach, certain ethical issues attached to data can be flagged, evaluated, and provided to all users and redeployed transparently. The following discussion addresses factors to be considered in creating an EAISIS in the development of an autonomous vehicle (AV). As a novel approach to AIDC, the issues raised are intended to serve as a framework for later discussion, research, development, and application.

## Sustainable artificial intelligence

### Sustainability

**Environmental** sustainability is grounded in respecting the environment, properly balancing between performance and cost, while maintaining social attention and care [2]. We assert that this model allowing economic growth, innovation and conservation of resources is also applicable to data and AIDC. Prior to engaging AIDC as the digital alternative to the traditional AV product life cycle of raw materials, processing, pre-assembly, assembly, distribution and end of life disposal, an introduction to AI is helpful.

To begin, an avenue for understanding and demystifying AI is to break down the term ‘Artificial Intelligence’, a term that often gets different interpretations depending on their user and their interests/goals [3]. An artefact used in the context of AI is something non-naturally occurring made by humans. Intelligence denotes the ability to make the right decision at the right time within a certain context [4]. Artificial versus natural intelligence is therefore simply a difference of origin. Like its organic counterpart, AI can make decisions based on observable information or data. With imperfect context and AI and machine learning algorithms can execute the appropriate function, or ‘right’ decision, with potentially damaging consequences [5]. For example, assume an intelligent agent was instructed to feed a stranger’s pet. Although seemingly simple, neither human nor machine can perform this task without having prior heuristics or additional data. Suppose the agent is informed the pet is domesticated, has four legs, requires regular feeding, and is located in North America. Given these data, certain assumptions can be made, resulting in certain possible animals to be eliminated, reducing the search space. While a turtle and a dog both fit the description, confusing their care instructions would be disastrous. These are the types of issues that arise when relying on bad or incomplete data.

### Artificial intelligence

Human mistakes and erroneous data impact AI functionality. Even when ultimately functioning completely autonomously, human decisions concerning training and/or test data are necessary in the AI development process. The ability of a machine, either carbon or silicon, to make a good decision is strongly related to the volume, statistical representation, and overall quality of these data provided within a given context. However, with accurate or unchallengeable data and a sufficiently narrow context and set of goals, AI can outperform even the most exceptional humans at certain tasks [6] (pp. 94–136).

This ability is astounding, but it must also be understood that algorithms date back to 2500 B.C. and are something used by people in everyday interactions [7]. An algorithm is simply a set of instructions to be followed in a certain sequence in order to achieve some outcome. For example, cooks regularly create and execute algorithms when passing or making family recipes. Given sufficient data, AI at its core is an instructional guide for the program’s actions within a certain context. However, like the recipe, inserting the wrong ingredients (data) or processing it incorrectly can result in a cake ranging from inedible to toxic for human consumption. Similarly, given the ‘bad data’, either due to negligence or maliciousness, an autonomous system can end up propagating biases [8], spreading misinformation, or reducing accuracy and robustness of the system [9].

### The digital environment

It is time to recognize that the divide between the digital environment and real life is largely illusory. Globally, the internet penetrates 58% of the population with a current growth rate of over 1000% and accessibility in all countries [10]. With the majority of countries employing shelter in place orders, currently or in the recent past, reliance on the digital environment has shifted from a convenience to a survival necessity. When the global COVID-19 pandemic threat arose IRL, people retreated to the safety of their ever-expanding colonies in the digital new world. Even prior to this, the digital environment supplied educational resources, coordinated travel, and assisted with finding dining decisions for friends and spouses [11]. AI and digital growth depend on IRL resources to meet their voracious appetite for data and electrical power consumption [12]. As pioneers in the new digital frontier, the trailblazers owe a duty of care to the next generation. Humans have spoiled every frontier they have encountered: littering or contaminating land, sea, air, and space [13]. Through ignorance—or avarice—this cycle continually repeats. Although a plan for ethically sustainable AI may not solve all problems, ignorance should not be a valid excuse to repeat the past.

Prior to discussing digital resource preservation for a sustainable AI, an important distinction between a virtual agent and its physical instantiation should be made. A virtual agent is a computer program, whilst a robot is a conduit requiring sensors, motors, and computational resources allowing the algorithm(s) to run, i.e. it is the combination of software and hardware.^1^ Without the physical embodiment of sensors and motors (more strictly actuators), an AI-based system’s direct physical influence IRL is limited. The remainder of this paper focuses on AI as located in the digital environment of the internet.

## Ethical artificial intelligence sustainability impact statement

This section proposes factors and stages for developing a sustainable digital environment for AIDC. While focusing entirely on the data needs of AI, at all times humans are the intended beneficiary of this analysis. The aim of this section is to identify data toxicity and its potentially negative impact on human end users and IRL resources.

### Artificial intelligence developmental cycle

The artificial intelligence developmental cycle (AIDC)^2^ model approaches data as a limited resource that is critical for AI development. As AI is intended to help all of humanity, a sustainable data approach should be considered a public good. Toward this end, the AIDC requires accepting the following principles:

- Humans are the moral patient. AI is a tool meant to serve humans. Artificial Intelligence functioning should only occur in the absence of or to protect against threats to the safety and welfare of humans [14].
- Intelligent systems are a product [15]. Although an autonomous tool, AI is still *only a tool. AI bears no more responsibility or moral accountability for negative consequences of fulfilling its programmed functions than a toaster can be found guilty of burning bread.
- An intelligent system should never be unconditionally, or naively, trusted [16]. As an artefact produced by humans, due diligence should include considerations of human malfeasance, misfeasance, motivations, and moral practices. This includes questioning the materials employed and processing employed during the AIDC.
- AI exists in the digital world, but impacts IRL.
- The digital world is a shared resource requiring certain protections for the common good.
- Sustainable AI is threatened by scarcity of resources (data) in the digital environment.

Grounded in these assumptions, the following stages are proposed for sustainable AIDC discussion.

### AIDC stages

Data are a foundational and critical component for producing AI. The creation, functioning and future adoption of AI depend on quality data. Defined as ‘factual information (such as measurements or statistics) used as a basis for reasoning, discussion, or calculation’ by Merriam Webster, it informs AI and people alike. However, invoking the first commandment of machine learning: ‘garbage in, garbage out’ [9], it is apparent the value of data needs to be accurate, consistent, and contextually appropriate for proper machine functioning.

#### Stage 1: Dumb data acquisition (raw material and sourcing)

Data acquisition for AI shares many characteristics with the more traditional manufactured product life cycle. However, the unique nature and specifically tailored needs of data for machine learning require a novel and hybrid approach for this new commodity. Currently, the data brokerage industry is fast-growing with data brokers desperately attempting to supply the insatiable appetite for machine learning [1]. As the industry expands exponentially, demand on IRL for resources similarly increases. The cost in human labour exploitation and natural resource consumption to support the head-spinning pace of this disruptive technology is sobering [17]. While recognizing the potential harmful impacts and IRL costs to the environment and disadvantaged populations, this sustainability analysis focuses on the data itself as a sustainable product. As demand grows, so will the commodity price of data, resulting in an increased likelihood of inadvertently acquiring toxic data. This will likely follow the same pattern as other industries. Secondary derivative markets will form to trade a debased and inferior version of the original commodity at lower prices, in this case contaminated data. With players racing to market whilst keeping the costs low, certain aspects will have to be prioritized and others will be ignored. Security in these scenarios draws the short straw, and your privacy is the cost [18].

Whether processing new data or reusing established data, the following ethical concerns should be considered during **Stage 1 **data capture:

Ethical Identification:

- Are identification and labelling processes conducted in a manner free from undue influence or manipulation?
- Have identification processes considered applicability to the digitally vulnerable?^3^
- To what degree is identification robust and universally applicable?^4^
- Is identification translatable and interpretable cross-culturally?
- If personal data is involved has it been properly privatized according to a set metric?

Ethical Capture:

- Were data freely observable ‘in the wild’, requiring no personal information, consent or individual interaction for capture?
- If consent was required, was it obtained in a manner consistent with local, national and international laws and regulation?
- Was consent given through digital duress under a ‘take it or leave it’ adhesion contract, possibly subject to later challenge?
- Were the intellectual property rights of the original data creator/compiler/subject respected through appropriate compensation or other sufficient consideration?
- Has transparency regarding data origin through chain of commerce been properly documented and maintained?

Toxicity Level:

- The above serve as framing questions for an in depth EAISIS. The varied end user sophistication, local regulations, intended use and need to protect naive users must also be factored in any analysis.

*We shall make use of autonomous vehicles (AVs) a running example throughout.* AVs must depend on ‘dumb data’ at the foundational level, to form the basis of the maps that the sensors will read to understand the layout of the road, obstacles that are permanent, general rules of particular roads (including, for example, speed limits, whether a right turn is allowed, et cetera). This is all before any smart navigation is conducted.

#### Stage 2: Domestication training for smart data

The previous stage focuses on data as a raw material acquired through capture in ‘the wild’ or possible reuse. This section addresses the preparation and use of the data in a sustainable manner for future reuse. The process of refining captured or brokered ‘Dumb Data’^5^ to ethically filtered ‘Smart Data’^6^ for AI training and future use raises many significant issues. As the data are deployed within functional programs, it has yet to be customized beyond manufacturer settings. During this portion of AI training, the programmer represents the greatest ethical concerns for data manipulation or misuse. Consider the following:

- To what degree do we consider the bias, motivations, or simple skill level of the programmer?
- Should a user’s decisions be limited by a programmer’s judgement?
- To what degree will decisions with moral weight be automated by factory presets?
- Are programmers’ ethical decisions incentivized by corporations?

These questions will require further exploration in an EAISIS analysis of the AIDC. These types of questions are best addressed and weighed for impact on sustainability during this stage. The different stages of machine learning coincide with the developer’s level of dominion and control over the application’s use and exposure to outside data, or ability to maintain a controlled environment. Prior to acquiring user specific preferences through human machine interaction, programmers are preparing it for a specific application for targeted users. However, no amount of simulation will sufficiently inoculate the program to the many new variables that will likely result once under control of the distributer or end user. During this phase of the AIDC, the AI is under complete control of the manufacturer or developer. The data employed for training and machine learning should be in its best form for its intended purpose, hold a toxicity rating and maintain a clear record detailing the chain of custody prior to its use for development. Although a seemingly relatively secure refuge from the risk of toxification, this stage is ethically challenging for the programmers. An Accountable, Responsible and Transparent Artificial Intelligence (ART-AI) ethical analysis framework brings some of these issues into focus:

Accountability:

- Was a record of AIDC leading to this point accurately maintained to identify liable party(ies) for product-related injuries?
- What guarantees or safeguards are in place to assist consumers in seeking help and identifying a human to hold accountable?
- What steps can be taken to minimize embedding conscious and unconscious bias in programming and data refinement?
- How will this application and use of data impact individual or group human rights?
- What information can be ascertained, either directly or indirectly through processing, about the original data source that may be outside the original consent of the source?

Responsibility

- Are safeguards in place, including insurance or other monetary funds to compensate for product defects or to mitigate damages?
- Have the programming and design team maintained a model for continuing education on ethical Artificial Intelligence implementation?
- Has an independent outside body or suitably trained and designated Ethics Officer conducted a thorough EAISIS?
- Are sufficient resources dedicated to educating and assisting the public regarding risks of AI and data sharing?
- Whose job is it to ensure the original privacy guarantees are maintained throughout the lifecycle?

Transparency

- Can the AI algorithmic decision making be monitored and explained to the consumer?
- Where was the origin of the data and how has it been changed?
- When were the data captured, and over what time interval?
- Why were the data captured, and is this use in line with that original purpose?
- Will explicit consent be obtained from ongoing user data capture during use of product?
- What safeguards are in place to avoid AI deceiving naïve users through deliberate or accidental confusion or anthropomorphized emotional manipulation?
- Is the chain of command and responsibility for any product defect clear and accessible to the public?
- Has the toxicity level been disclosed conspicuously and clearly?
- Have factory set AI motivational settings and risks to the consumer been clearly disclosed?

As the AI moves through AIDC, the levels of future toxicity will increase as the trained applications are released back into the wild. These stages hold different threats for the ethical sustainability of AI. During Stage 3, the AIDC enters the period of greatest learning potential and risk of toxicity.

AVs will use sensors to read maps of pixels, and objects which are identified in the data collected will be used for navigation. There is no guarantee that such objects will be present in the dumb data that is collected. As such, AVs will be vulnerable to unconscious biases from programmers, such as the ways that a particular programmer drives and the rules of the road that are customary in that programmer’s country or part of the world. Furthermore, AVs will have access to the data which is selected by programmers as relevant for use. At some stage, an AV must prioritize one outcome over another: safety, efficiency, and cost will likely be some of the primary factors.

AVs will also be vulnerable to conscious manipulation; for example, governments may, in the name of safety, restrict AVs from driving above the speed limit in a given area. The eventual homogenization of vehicle speeds through the adoption of AVs as the most common form of vehicle presents as a positive outcome on its face. However, individual drivers may have valid reasons for increasing speed, and the removal of that choice through the strict enforcement of law could lead to negative outcomes. Consider, for example, a car coming into an occupied lane. The car which is already in that lane may honk its horn, slam on its brakes, or even speed up to get out of the way before a collision happens. Removing the option of speeding up could lead to accidents which would not have otherwise occurred.

#### Stage 3: Return to the wild and the ecosystem of trust

AI is meant to benefit and serve humans through the ability to learn and make decisions autonomously. Placing AI in a consumer’s hands accelerates this process. This is when AI learns ‘street smart’ lessons, both beneficial and toxic. The utility of AI depends on its ability to learn from these lessons and offer consistent, accurate and predictive solutions for the user. Mass adoption and the ability to maintain AI as an embedded and reliable resource rely on maintaining good data for this purpose. Sustainable AI depends on maintaining quality data through an ecosystem of accuracy and transparency.

As this portion of conducting an EAISIS is largely speculative, this early in the process of AI and AV adoption, the missteps of social media can teach us some lessons about maintaining public trust. At the forefront of establishing trust in any interactive technology, the consumer needs reassurance against invasion of privacy, inaccurate information (fake news) and potential manipulation. The success and accuracy of predictive systems to make the right decision for each user depends on candour. Largely depending on self-reported or observed behaviour, AI requires true and accurate data to function properly. This requires trust and confidence to reveal personal information.

Consumer trust is continually eroded by data breaches, ransomware attacks, improper digital information capture and so on. This combined with other bad faith actions by companies undermines already shaky corporate trust. The result is a catch-22 scenario wherein trust in the corporate is needed to establish trust in the product, but the big data companies largely squandered what little they previously held [19].

During Stage 3, the AI has the greatest ability to learn the appropriate response for a given context based on observations. This is not a new practice, with most internet users encountering personalized dynamic advertisements and targeted news feeds daily, if not hourly or even more frequently. These are created through combinations of data mining, web scraping, emotional recognition, and locational monitoring amongst other factors [20]. Although seemingly intrusive and despite protests of data gathering over-reach, it is a simple barter system. Data of commercial value are traded for a service or convenience of personal value.^7^ Increased use of domestic robots has also increased opportunities for data capture.

As the novelty of owning a robot, like Alexa, wanes and dependency on it as a digital resource increases, familiarity of use can provide domestic robots candid and intimate information about their users [21]. This will most likely be compounded with AV. Drivers can imagine themselves as invisible when travelling alone, often inadvertently singing, dancing or practising even more private habits in public view.^8^ An ongoing EAISIS model allows users security in knowing the fate of their sensitive personal data reinforcing the ecosystem of trust required for sustainable AI.

As AI and AVs become more widely adopted, the EAISIS should ask a series of questions to determine what tests and controls have been put in place to ensure that the AI is behaving sensibly. While additional questions will likely develop over the course of EAISIS onboarding and widespread use, some questions for consideration include:

- What sorts of common scenarios have been identified, and what tests have been established and carried out to ensure that the AI acts as it should in such scenarios?
- What controls have been put in place to mitigate harm to humans if AI is not used properly?
- How does the AI correct for deficiencies in its user base? In particular, the age of the user might lead to unexpected decision making, especially when the user is a child or quite elderly. Can the AI detect when a user is making illogical choices and correct for them?

In the case of AVs, road tests for licensing allow for common scenarios to be presented. However, manufacturers must balance protection with an individual’s ability to override the AI.

#### Stage 4: Toxic data mining: remove, retire, rescind, redistribute, or redress

The final stage of AIDC relies on the previous toxicity rating and evaluation for future human use. The existing toxicity is determined by levels of inaccuracies, social unacceptability, or danger to human interests. These factors weighed against the ability to and difficulty in removing toxicity can be used to determine future reuse. The acceptable level of data toxicity will depend on the intended future use and foreseeable danger for unsophisticated end users. The following mitigation approaches are suggested:

- *Remove:* Removing data should remain a decision for the supplier of the raw material, data. Currently, the Right to Be Forgotten from the Google Spain Case [22] and the recent findings against Volkswagen for DeiselGate [23] are paving the way for legal avenues to invoke this right.
- *Retire:* Rather than outright removal or deletion, personalized data can be placed in a data trust for transferability to other smart devices allowing portability of personalized systems.
- *Rescind:* As discussed, data capture is largely a barter system, wherein data is traded for a digital service or product. The internet is forever but the conditions of a barter contract change. Rescission allows users to limit the unauthorized exploitation of their data.
- *Redistribute:* As AI is adopted and learns individual or culturally specific lessons, these heuristics can be helpful to others wanting to train their machines similarly. For example: One AV driver may place greater weight on the risk of being stranded alone at night over the fuel and time savings of travelling on sparsely populated roads. These types of heuristics and personalizations should be capable of redistribution. Intellectual property protection should also be extended for any unique approaches to algorithmic reasoning that may become widely adopted and potentially profitable.
- *Redress:* Data are a commodity, and data creation for future use should be compensable. If personal data are employed for commercial enterprise, the originator should receive payment to reduce future liability claims or other challenges to its legitimate capture. Alternatively, if personalized data are mishandled resulting in harm to an individual, a standard system for redressing grievances, including a well-defined compensation structure based on liquidated damages clauses, should be considered.

Employing one or a combination of the above methods to detoxify data would allow capture of valuable information while protecting personal interests. Remembering the goal of maintaining data in a manner free from human harm, if successfully applied an EAISIS approach can preserve useful data while reducing toxicity. However, care must be applied, with data being used, modified, reused and redistributed the source may become fuzzy and change altogether. Any of these mitigations may be applied to the current state of the data, however it may not be enough. If the data being acted upon are a direct derivative, either by modification or redistribution of another data source, should its derivatives and original roots be treated the same way? This scenario has deep-rooted influences in how data ownership is discussed and in how the legal agreements between distributors and consumers can be constructed. To properly address these issues, we propose that clear chains of origin, custody and modification be in place from original data through each of its consequent evolutions to ensure proper regulation and ethical considerations. This could easily be achieved transparently using blockchain technologies or any other auditable data structures.

## Conclusion

The mass adoption and increased demand for data require a sustainable approach for AIDC. Reducing demands on natural resources and human labour and maintaining a digital environment capable of supporting a healthy, reliable, and safe AI ecosystem is a public good. The risk of integrating bad data in an AI application may result in small errors like recommending the wrong dining option, or more existential risks of AI misinterpreting data to disastrous effects [24](“SUPERINTELLIGENCE” 85).

The historical missteps that have led to the current environmental decay and toxicity of shared resources must be addressed, and the same mistakes must not be repeated in the digital world. As pioneers in this new realm, we have a responsibility to maintain the digital world in a sustainable manner for future generations. The Ethical Artificial Intelligence Sustainability Impact Statement, through the various stages of the AIDC, serve as a springboard into discussion regarding the future of a sustainable digital environment.

## Appendix: Right of the child summary, by Julia Bogani

The new expansion of Artificial Intelligence(AI) and the accompanying data within the past 10 years has called for some regulations. Like any new thing, Artificial Intelligence, such as Alexa, Siri,and Google Assistant, must have some ethical (what’s right and wrong) restrictions before it becomes a major problem in society. This is a proposal for an Ethical Artificial Intelligence Sustainability Impact Statement throughout the AIDC. These questions are intended to identify and address the data’s potential to be harmful. The data’s potential harm can have negative impacts on human users, including children, and resources unless there are regulations such as these in place.

The [proposed] AIDC has four stages: (1) Dumb data acquisition, (2) domestication training for smart data, (3) return to the wild and the ecosystem of trust, and (4) toxic data mining. Dumb Data Acquisition addresses data found through capture and potential reuse. The second stage, Domestication Training for Smart Data focuses on using data in a preserved manner for future use. Return to the wild and the ecosystem of Trust discusses public trust and privacy. The final stage would help to allow ownership of valuable user information, while protecting personal interests. These stages are important to the development of ethical future AI. The issues addressed are intended to act as a framework for future discussion, research, development, and application of AI in future systems.

## Footnotes

1 This is known as a cyber physical system, namely the conjunction of real-world mechanisms monitored by a digital component. We reserve further discussion of physical implication of unethical data usage for future work.

2 Recognizing the ever-present threat of anthropomorphism, the term ‘life cycle’ should be prohibited with AI.

3 Those individuals or groups of people lacking sufficient or equal access to the internet regardless of cause, at times called the ‘digitally invisible’.

4 Universality allows easier transfer of resources and ability to advance the digitally vulnerable and narrow the digital divide.

5 Data freely observable in the public domain and holding no legal or moral deficiencies of title (raw data).

6 After Processing/Subassembly/Assembly/Pre and Post Distribution.

7 Recognizing many issues exist regarding duress or adhesion contracts, further discussion is outside the scope of this paper. See ibid.

8 Such as nosepicking: https://www.healthline.com/health/nose-picking.

## Declarations

### Supplementary information

In acknowledgement of UN Rights of the child regulations we provide child friendly summary of this work in Appendix, by Julia Bogani.
